# B lymphocytes can be activated to act as antigen presenting cells to promote anti-tumor responses

**DOI:** 10.1371/journal.pone.0199034

**Published:** 2018-07-05

**Authors:** Renata Ariza Marques Rossetti, Noely Paula Cristina Lorenzi, Kaori Yokochi, Maria Beatriz Sartor de Faria Rosa, Luciana Benevides, Paulo Francisco Ramos Margarido, Edmund Chada Baracat, Jesus Paula Carvalho, Luisa Lina Villa, Ana Paula Lepique

**Affiliations:** 1 Department of Immunology, Instituto de Ciências Biomédicas, Universidade de São Paulo, São Paulo, Brazil; 2 Hospital Universitário, Universidade de São Paulo, São Paulo, Brazil; 3 Instituto do Câncer do Estado de São Paulo, Faculdade de Medicina, Universidade de São Paulo, São Paulo, Brazil; 4 Department of Immunology and Biochemistry, Faculdade de Medicina de Ribeirao Preto, University of Sao Paulo, Ribeirão Preto, Brazil; 5 Instituto de Radiologia, Faculdade de Medicina, Universidade de São Paulo, São Paulo, Brazil; Mie Daigaku, JAPAN

## Abstract

Immune evasion by tumors includes several different mechanisms, including the inefficiency of antigen presenting cells (APCs) to trigger anti-tumor T cell responses. B lymphocytes may display a pro-tumoral role but can also be modulated to function as antigen presenting cells to T lymphocytes, capable of triggering anti-cancer immune responses. While dendritic cells, DCs, are the best APC population to activate naive T cells, DCs or their precursors, monocytes, are frequently modulated by tumors, displaying a tolerogenic phenotype in cancer patients. In patients with cervical cancer, we observed that monocyte derived DCs are tolerogenic, inhibiting allogeneic T cell activation compared to the same population obtained from patients with precursor lesions or cervicitis. In this work, we show that B lymphocytes from cervical cancer patients respond to treatment with sCD40L and IL-4 by increasing the CD80^+^CD86^+^ population, therefore potentially increasing their ability to activate T cells. To test if B lymphocytes could actually trigger anti-tumor T cell responses, we designed an experimental model where we harvested T and B lymphocytes, or dendritic cells, from tumor bearing donors, and after APC stimulation, transplanted them, together with T cells into RAG1^-/-^ recipients, previously injected with tumor cells. We were able to show that anti-CD40 activated B lymphocytes could trigger secondary T cell responses, dependent on MHC-II expression. Moreover, we showed that dendritic cells were resistant to the anti-CD40 treatment and unable to stimulate anti-tumor responses. In summary, our results suggest that B lymphocytes may be used as a tool for immunotherapy against cancer.

## Introduction

Human Papillomavirus is the main etiologic factor for cervical cancer and a percentage of other anogenital and oropharyngeal cancers [1,2]. The natural history of cervical cancer is long, involving molecular and cellular alterations, as well as immune evasion [3,4]. Both effector and regulatory T lymphocytes infiltrate cervical tumors, where low effector/regulatory T cell ratio is a poor prognostic factor for disease progression and metastasis [5]. Systemically, it has been observed that circulating T cells from cervical cancer patients preferentially exhibit regulatory phenotype, with low proliferation and IL-10 secretion upon stimulation with HPV antigens, indicating that these tumors are capable of inducing tolerance [6].

Interestingly, de Vos van Steenwijk and collaborators have shown that patients with cervical cancer display a surprisingly large, although inactive repertoire of T cells that recognize the viral E6 and E7 HPV antigens [7]. This is also the case with other types of cancer, where T lymphocytes can recognize tumor antigens [8]. This knowledge has prompted many research groups to investigate ways to break tumor induced tolerance and boost immune responses against tumor antigens. Among various approaches, the most recent ones are the use of anti-CTLA4, anti-PD-1 and PD-L1 antibodies [9]. Additionally, the autologous or allogeneic transfer of antigen loaded dendritic cells or antigen specific activated T lymphocytes to cancer patients has been widely explored [10–12]. Dendritic cells-based therapy is usually based on monocyte derived DCs, which involves *ex vivo* maturation and activation of DCs from monocytes. It has been related that monocytes from patients are susceptible to the tolerogenic mechanisms triggered by the tumor microenvironment, which has been frustrating researchers for many years [13–15]. Our laboratory has recently shown that monocyte derived DCs from cervical patients are tolerogenic compared to the same type of population obtained from patients with cervical intraepithelial neoplasia or cervicitis [16].

B lymphocytes are usually regarded as a pro-tumoral population. These cells can promote tumor progression through IgG secretion and activation of inflammatory cells, causing chronic inflammation [17,18]. B cells can also secrete cytokines to inhibit T cell activity, promote angiogenesis and cell survival within the tumor microenvironment or present tumor antigens generating regulatory responses [19]. Indeed, it has been shown that depletion of B lymphocytes may enhance anti-tumor vaccine efficiency, inhibit solid tumor growth and inhibit regulatory T cell expansion in mouse tumor experimental models [20,21]. In this sense, most anti-tumor therapies involving B lymphocytes suggest depletion or inhibition of this population. Drugs like Rituximab, a humanized anti-CD20 antibody and Ibrutinib, an inhibitor of Btk (Bruton’s tyrosine kinase), are possible candidates for such therapies [19,22,23].

Our laboratory has been investigating local and systemic tumor effects on cervical cancer patients’ immune system and HPV associated mouse tumor models. Using a murine tumor experimental model, it came to our attention that HPV16 tumor cell line, TC-1 [24], caused a significant increase in the absolute number of B cells in the tumor draining lymph nodes [25], which brought this population to our attention. Interestingly, in spite of the data indicating that B lymphocytes are usually a pro-tumoral population, there is also evidence that these cells can be modulated to promote anti-cancer T cell responses [23,26–28]. Specifically, regarding cervical cancer patients, aiming to study T cell responses against HPV antigens in cervical cancer patients, van der Burg and collaborators have successfully made use of a mix of EBV (Epstein Barr Virus) immortalized B lymphocytes, which included B cells from cervical cancer patients, to present antigens to T cells. This work indicated that B lymphocytes from cervical cancer patients were able to present antigens to T cells in *in vitro* activation assays [6]. Therefore, we decided to investigate whether B lymphocytes could be used, under a therapeutic perspective, as antigen presenting cells and able to stimulate anti-tumor responses. We used *ex vivo* activated B lymphocytes to stimulate secondary anti-tumor T cell responses in a HPV16 tumor experimental model and were able to show that B lymphocytes may be refractory to tolerogenic mechanisms triggered by HPV associated tumors and may promote anti-tumor T cell responses. Our results suggest that B cells should be considered as an anti-tumor therapeutic tool.

## Materials and methods

### Patients’ cohort

Patients with cervical cancer referred to Hospital Universitário, Universidade de São Paulo, or Instituto do Câncer do Estado de São Paulo, ICESP, were enrolled in this study. Patients with any kind of immunodeficiency, immunosuppression or pregnant women were excluded from the study. We harvested 10 ml of peripheral blood from each patient previous to the procedure to excise the cervical lesion. All procedures involving patients were approved by the Ethics in Research Committee at the Institute of Biomedical Sciences, Hospital Universitário (process 1405) and the Committee for Ethics in Research at ICESP (10552412.0.3001.0076). We only enrolled patients that read and signed the Informed Consent Form to take part of the study. No patients under 18 years old were recruited to the study. The average age of our group of patients was 45 years old. All patients had cervical samples genotyped for HPV infection detection and virus type determination, and all of the cancer biopsies were positive to high risk HPV DNA (HPV genotype was determined using the Linear Array HPV Genotyping, Roche Molecular Diagnostics, Pleasanton, CA, according to the manufacturer’s instructions). We also harvested blood from healthy subjects, and these samples were used as controls. The age average of this control group was 40 years old, all of them were women and they were not tested for HPV infection. We used samples from 18 cervical cancer patients and 8 healthy donors.

### Peripheral blood mononuclear cells isolation and treatment with activating drugs

Peripheral blood mononuclear cells, PBMCs, were isolated through Ficoll-Paque gradient, counted and frozen in 10% fetal bovine serum, FBS, in RPMI and 10% DMSO (dimethyl sulfoxide, Sigma-Aldrich, St. Louis, MO). Cell viability was never less than 90% after density gradient isolation and after thawing for culture. Cells were counted, aliquoted and plated at the density of 10^6^ cells/well in a 24 wells culture plates in 10% FBS and RPMI. Cells were stimulated for 24 hours with sCD40L and IL-4 (Thermo Scientific, Waltham, MA, and Peprotech, Rocky Hill, NJ). Treatment doses were determined by literature references and dose-response curves that measured cell viability and expression of co-stimulatory molecules (S1 Fig). After treatment, cells were harvested, washed and incubated with antibodies against surface markers for flow cytometry analyzes.

### Antibodies

From BD Biosciences (Carlsbad, CA) against: mCD45 (clone 30-F11), mCD4 (clone GK1.5), mCD8 (clone 53–6.7), mCD40 (clone HM40-3), mCD80 (clone 16-10A1), mCD86 (clone GL1), mCD19 (clone 1D3), hCD45 (clone HI30), hCD19 (clone HIB19), hCD3 (clone HIT3a), HLA-DR (clone TU39), hCD80 (clone L307.4), hCD86 (clone 2331, FUN-1), and from eBiosciences (San Diego, CA): anti-mMHC-II (clone M5/114.15.2), anti-mFoxp3; anti-CD11b from R&D Systems (Minneapolis, MN). Antibodies against phosphorylated signaling proteins were purchased from Cell Signaling (Danvers, MA).

### Cell line and tumor

TC-1 cell line was kindly donated by Prof. Wu (John Hopkins Hospital, Baltimore, MA). TC-1 tumor model is well characterized and, more importantly, express HPV16 E6 and E7 oncoproteins, which allows us to study antigen specific responses [24]. To obtain tumors, we injected 1x10^5^ (donors for chimeras) or 5x10^4^ TC-1 (recipients for chimeras) cells subcutaneously per mouse in 100 μl of PBS^++^ (1mM CaCl_2_, 0.5mM MgCl_2_).

### Mice

For all experiments, we used 5 weeks old female mice of the following strains: C57BL/6, RAG1^-/-^ (B6.129S7-Rag1^tm1Mom^/J, The Jackson Laboratory, Bar Harbor, ME) and BKO (or muMt-, B6.129S2-*Ighm*^tm1Cgn^/J, The Jackson Laboratory, Bar Harbor, ME). They were all maintained in the Department of Immunology, Institute of Biomedical Sciences. The MHC-II^-/-^ mice (B6.129S2-Cita^tm1Ccum^/J) were maintained in the School of Medicine of Ribeirão Preto, University of São Paulo. Experimental and maintenance protocols involving animals were approved by the Instituto de Ciências Biomédicas Committee for Animal Use (process 2010/151).

### Immunization

C57BL/6 mice were intraperitoneally, *ip*, injected with 10 μg peptides E6_48-57_ and E7_43-77_ peptides (Genscript, Piscataway, NJ) with 100 μg ODN1826 (Thermo Fischer Scientific, Waltham, MA) twice, with a 15 days interval.

### Cell and tissue preparations

Single cell suspensions were prepared as described [29].

### Cell sorting

Once TC-1 tumors reached approximately 1 cm in the largest diameter, we euthanized the mice and harvested peripheral lymph nodes and spleen (at this point these tumors display all systemic effects on the immune system, leukocytosis, increase in B cell frequency in the lymph nodes, tolerance towards tumor antigens [25,30]). B and T lymphocytes were negatively selected from lymph node cell suspensions and lymph node and spleen suspensions, respectively (pan-B cell and pan-T cell isolation kits, Miltenyi Biotec, DE). For splenic DC isolation, we injected 200 μl of 1mg/ml Collagenase I and IV into the spleen of tumor bearing mice and incubated the spleens in a 37°C incubator for 30 min, prior to mechanical dissociation, followed by red cells lysis, and DC isolation using a Miltenyi kit for DC isolation (Miltenyi Biotec, DE). In all cases, we obtained 98% cell purity after sorting.

### Anti-CD40 treatment of murine cells

For all experiments, 10^6^ viable B lymphocytes or DCs/100 μl of medium were treated with 0.1 μg/ml anti-CD40 14h to16h at 37°C, except for signaling, where cells were treated for 30 min. Cells were washed twice with PBS and suspended in PBS^++^ at a concentration of 1 to 3 million cells together with the same number of T cells, to be injected per mouse.

### Chimera set up

Cells prepared as described above were injected *ip* in RAG1^-/-^ mice 5 days prior to transplant with 5x10^4^ TC-1 cells. The recipient mice were observed every 2 to 3 days for tumor measurements with a caliper.

### T cell activation assay

T cells from tumor bearing mice were labeled with CellTrace Violet (Thermo Fischer Scientific, Waltham, MA) and incubated in a 1:1 ratio with activated or control B lymphocytes, before harvesting, staining for surface markers and analysis by flow cytometry.

### Flow cytometry analyzes

Single cell suspensions were labeled with antibodies against surface or intracellular targets, as NFkappaB and ERK [31]. Labeled cells were analyzed in a FACSCanto (BD Biosciences, Carlsbad, CA), at least 30.000 events were acquired per sample. In all cases, doublets and debris were excluded from the analyzed events.

### Statistical analysis

Tumor growth kinetics were analyzed by the non-parametric Mann-Whitney U test and where indicated, we calculated the area under the kinetics resulting curves and compared the data by ANOVA; human B cell data was tested by ANOVA followed by Tukey pair analysis; mouse B cell data was tested Student’s t-test. In all cases, we considered p<0.05 as indicative that differences between groups were statistically significant.

## Results

### Circulating B lymphocytes from cervical cancer patients are activated by treatment with CD40L and IL-4

**Fig 1:**
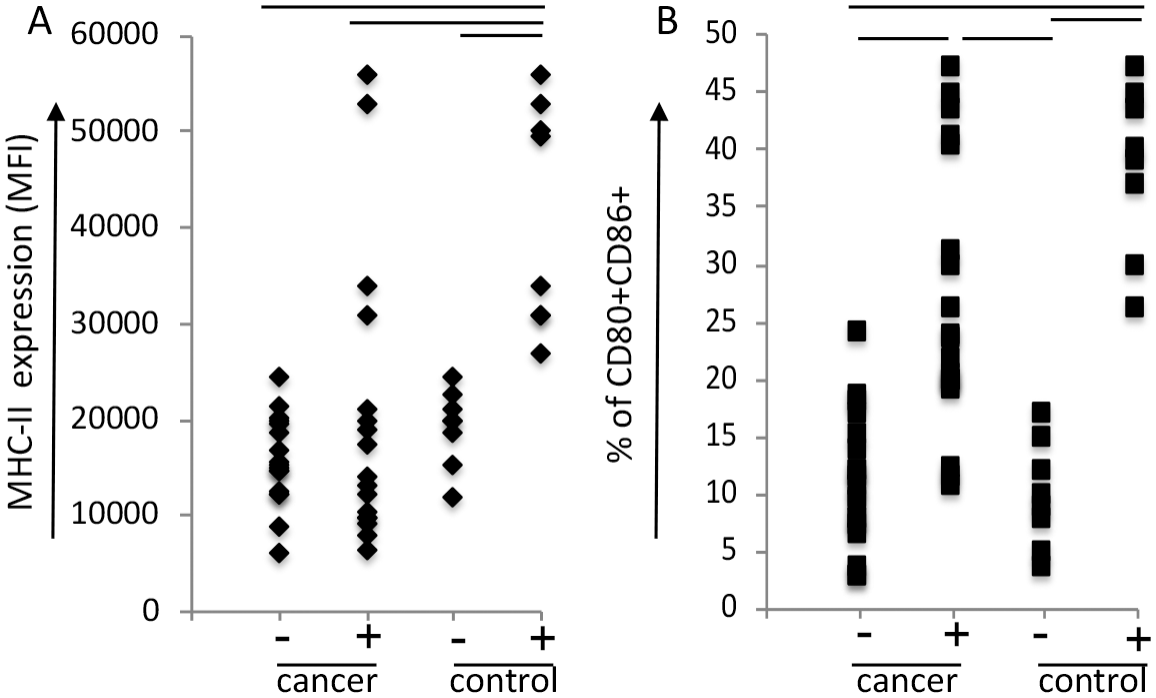
B lymphocytes from cancer patients can be activated by treatment with sCD40L and IL-4. Viable 90% enriched B lymphocytes from PBMCs from cervical cancer patients (cancer) or controls (control), were incubated with 10 μg/ml sCD40L and 50 ng/ml IL-4 for 24 hours. After harvesting, cells were labeled with anti-CD19, CD80, CD86 and HLA-DR and analyzed by flow cytometry, where 10^5^ events were acquired in a FACSCanto (BD Biosciences, Carlsbad, CA). For data analyzes, we excluded debris and doublets and gated the MHC-II, CD80^+^ and CD86^+^ populations on the CD19^+^ cells. We compared B lymphocytes from cancer patients (cancer) and controls (control), and within these two groups, sCD40L and IL-4 treated (+) and untreated cells (-). (A) Expression of MHC-II shown as median of fluorescence intensity (MFI); 100% of the cells were positive for MHC-II expression. (B) Percentage of the CD80^+^CD86^+^ population. Bars indicate groups with significant different results, based on ANOVA two-way test, where p<0.05 was elected as accepted. A total of 18 samples from patients with cancer and 8 samples from controls were used in this experiment.

Although DCs are the best activators of naïve T cells, there is evidence that monocyte derived DCs from cancer patients are poor T cell activators or tolerogenic [13–15]. As mentioned before, we have observed that in cervical cancer patients’ monocyte derived DCs are tolerogenic, significantly inhibiting allogeneic T cell activation [16]. However, B lymphocytes can activate secondary T cell responses if properly stimulated. Moreover, there is experimental evidence that immortalized B lymphocytes from cervical cancer patients can present antigens to T cells [6]. We, therefore, decided to investigate whether B lymphocytes from cancer patients could respond to stimuli, increasing its potential as an APC population. We studied the expression of CD80, CD86 and MHC-II on the surface of B lymphocytes from cancer patients, compared to cells from healthy donors. In healthy subjects, 10±4.6% of the B lymphocytes were CD80^+^CD86^+^ and stimulus with sCD40L and IL-4 increased this frequency to 38.4±7.2% of double positive cells. The HLA-II expression (present in virtually all B lymphocytes in both experimental groups), measured by median fluorescence intensity (MFI), displayed an expression increment of 2.2 fold upon treatment, from of 19±3.9x10^4^ to 41±11.7x10^4^. In cancer patients, we did not observe differences in the level of HLA-II expression upon stimulation, with average of 20.1±15x10^4^. The percentage of CD80^+^CD86^+^ cells, however, increased by a factor of 2.64 fold in cancer patients upon stimulation, while in healthy subjects this increase was 3.84 fold (Fig 1).

These results suggested that, although B lymphocytes from cervical cancer patients were not as responsive to IL-4 and sCD40L treatment as cells from healthy donors, indicating at least some systemic modulation of the tumor over this population, they were still able to respond to stimulus. We did not observe alterations in the frequency of naïve or memory B lymphocytes in cancer patients compared to control subjects, also indicating that cervical cancer does not cause major changes in the B lymphocyte populations (S2 Fig). These results led us to try to investigate, in an experimental model, the possibility of using B lymphocytes as antigen presenting cells to activate anti-tumor T cell responses.

### Tumor systemic effects suppressed T cells in a HPV16 positive experimental model

To test the possibility of using B lymphocytes as APCs, we chose a well described murine tumor model, the TC-1 HPV16 positive tumor cell line [24]. Several research groups use this tumor model, which has important characteristics as 100% efficiency in tumor formation in C57Black/6 mice [24], induction of systemic effects on the immune system [29,30], and previously described CD8 and CD4 recognized epitopes [32].

The immune system of C57Black/6 mice recognizes TC-1 tumors, although it is clear it cannot effectively control tumor growth. We inoculated wild type mice, RAG1^-/-^ mice and BKO mice (B lymphocytes deficient) with 1.5x10^4^ TC-1 cells and observed the tumor growth kinetics. As we injected a sub-optimal number of cells, we were able to observe differences in tumor growth in the different mouse strains. Tumors displayed the same growth kinetics when injected in wild type and BKO mice, however, they grew significantly faster in RAG1^-/-^ mice (S3 Fig). These results suggested that, while B cells did not influence tumor growth, T cells were important for containing it.

**Fig 2:**
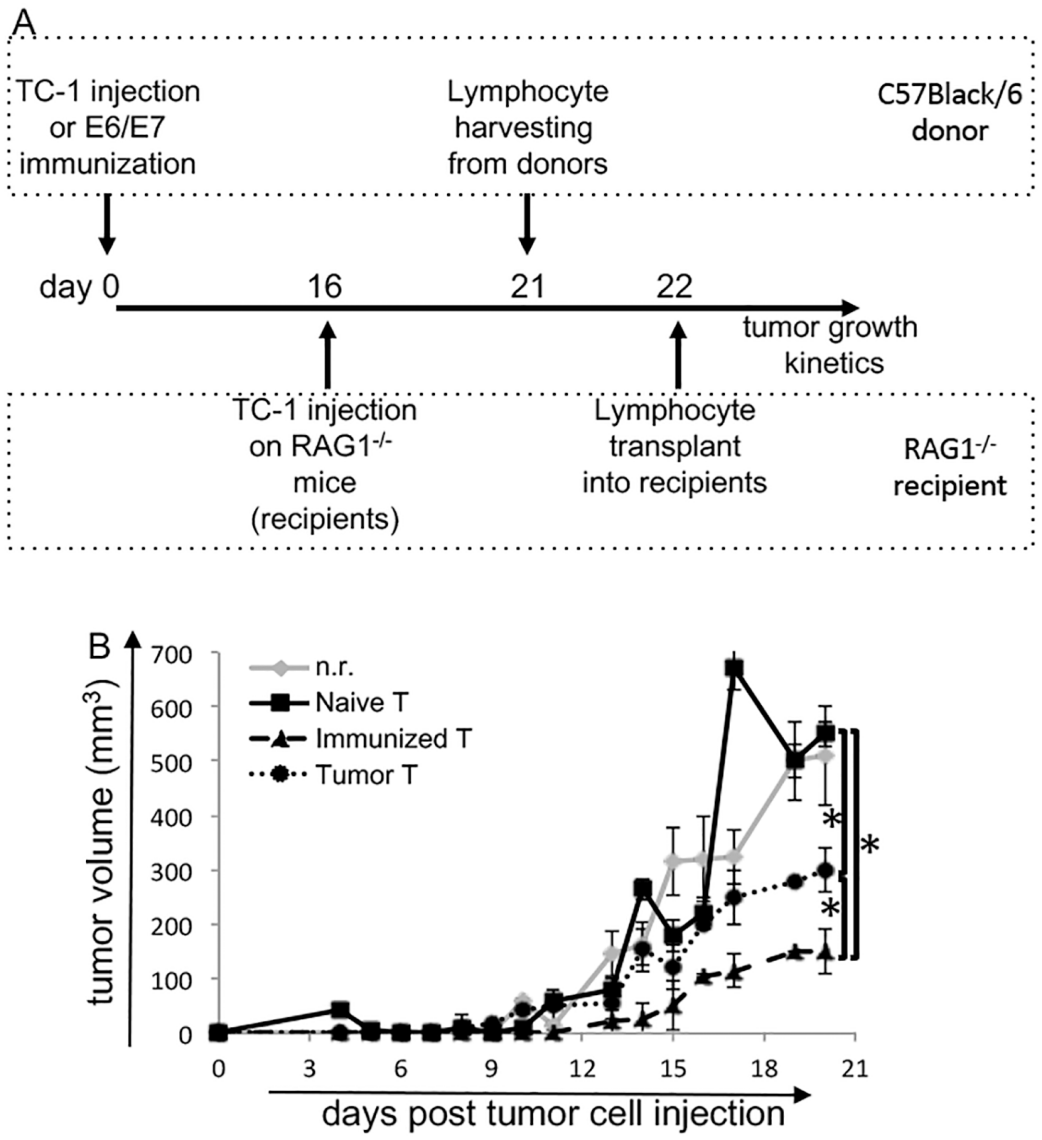
Tumors induce tolerance in secondary lymphoid organs, but not through B lymphocytes. (A) Diagram of the experimental design, where we harvested cells from C57Black/6 donors: naïve, with TC-1 tumors or immunized against tumor antigens; and transplanted into RAG1^-/-^ mice previously inoculated with TC-1 cells. After transplant, we measured tumor growth every other day for up to 21 days. (B) Tumor growth kinetics in RAG1^-/-^ mice transplanted with T lymphocytes isolated from lymph nodes from naïve, (Naïve T), HPV16 E6 and E7 peptides immunized (Immunizaed T), or TC-1 tumor bearing (Tumor T). nr—not reconstituted. One to 3 million lymphocytes were transplanted per mouse. Experimental groups of at least 6 mice (the experiments were repeated 3 times, so a total of at least 18 mice were tested); * indicates p<0.05, tested by Mann-Whitney U test. Experimental groups of at least 4 mice; * indicates p<0.05, tested by Mann-Whitney U test. We observed similar results when comparing the kinetics using the area under the curve as data for ANOVA testing.

To test the interaction between lymphoid populations in this experimental model, we designed an experiment model where we transferred T and B lymphocytes or DCs from C57Black/6 mice to TC-1 tumor bearing RAG1^-/-^ mice. The former is a non-leaky immunodeficient model, where no mature B or T cells are found due to deletion of the RAG1 gene [33]. The experiment consisted of injection TC-1 cells into RAG1^-/-^ mice, and five days later, time enough for the cells to graft into the recipients, we adoptively transferred lymphocytes from C57Black/6 donors (Fig 2A). In the first set of experiments, we transferred T cells from naïve, tumor bearing or anti-E6/E7 immunized C57Black/6 mice, which are isogenic to the tumor cells. We observed that in recipients transplanted with T cells from naïve donors, the tumor growth kinetics was the same as in non reconstituted recipients, n.r. (Fig 2B, figure legend indicates the source of T cells in each experimental group). T lymphocytes from immunized donors controlled tumor growth, and to our surprise T lymphocytes isolated from tumor bearing donors also, partially inhibited tumor growth. Our results suggested that TC-1 tumors controlled the immune responses in peripheral lymphoid organs microenvironment, and that by isolating T cells we could partially reverse the suppressive effects of the tumor on their activity.

### *Ex vivo* stimulated B lymphocytes activate T cells *in vitro*

**Fig 3:**
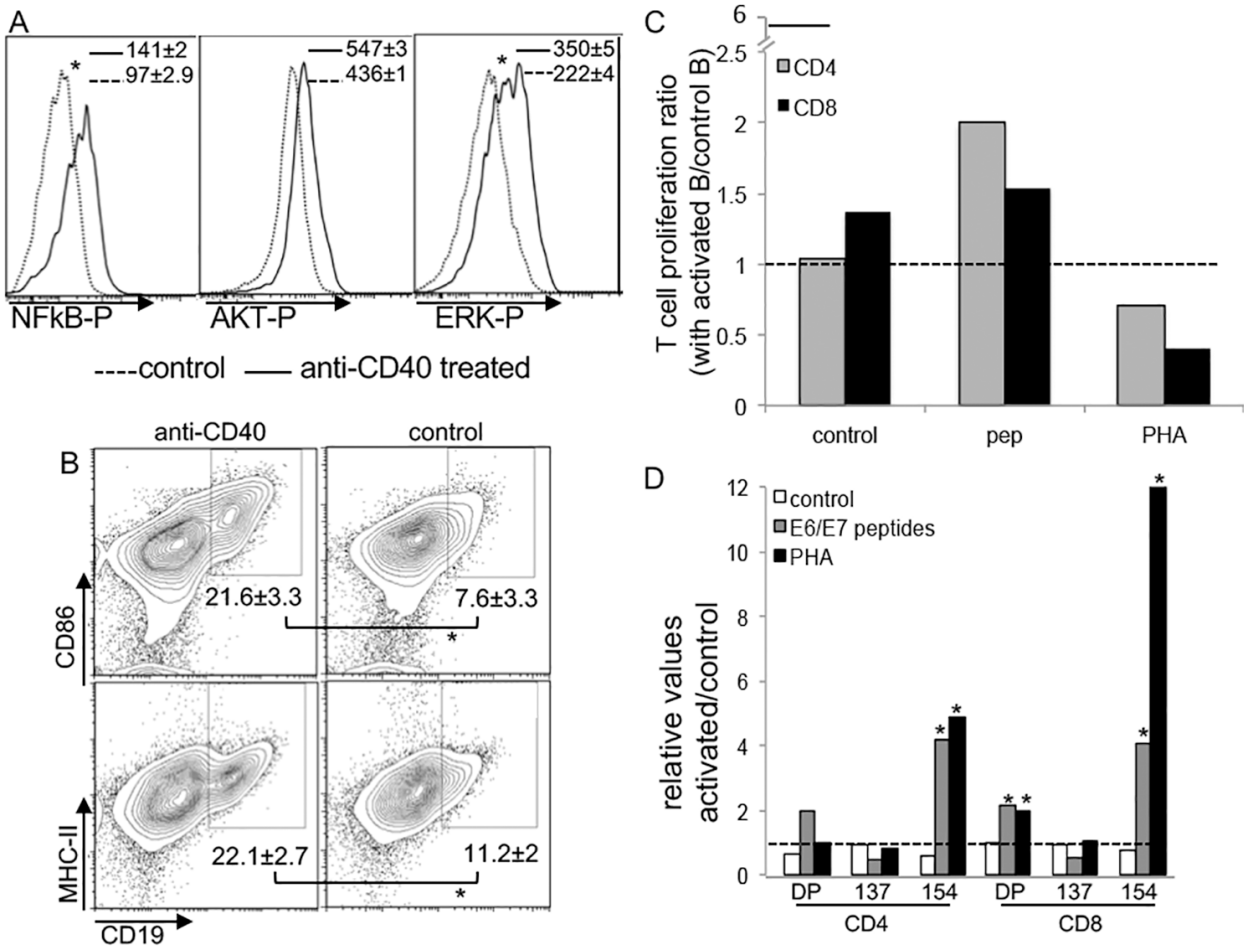
Activation of B lymphocytes with anti-CD40. (A) B lymphocytes sorted from tumor bearing C57Black/6 mice were treated *ex vivo* with 100 ng/ml anti-CD40 and analyzed by intracellular staining against the indicated phospho-proteins and flow cytometry; cells were stimulated for 30 min prior to harvesting. Results are the average of median fluorescence intensity of 3 independent experiments (indicated); (B) analysis of the expression of activation markers in peripheral lymph node cell suspensions determined by flow cytometry after 14 to 16 hours treatment. A representative experiment of 3 independent ones is shown (each experiment with at least 3 mice per group), and the average and standard deviation of the frequencies of the double-positive populations is shown in each dot-plot. Differences between control and treated cells were tested by t-test, * indicates p<0.05. (C) and (D) CellTrace labeled T lymphocytes from tumor bearing donors were incubated with sorted B lymphocytes from tumor bearing donors treated or not with 100 ng/ml anti-CD40 for 16 hours, in a 1:1 ratio. Phytohemagglutinin, (PHA) (1:100), or 5 μg/ml HPV16 E6 and E7 peptides (pep), were added to the cultures. Four days later, cells were harvested labeled with antibodies for CD4, CD8, CD154 and CD137 and analyzed by flow cytometry. The ratios of the percentage of CellTrace dim cells from cultures with anti-CD40 treated and control B lymphocytes (C) and expression ratio of CD154 or CD137, or both (DP) in cultures with treated and control B lymphocytes (D) are displayed. Ratios of three independent experiments, each one in biological triplicates (cells from 3 different mice, and our cultures were also plated in triplicates). The dashed lines indicate ratio value 1, where activation was the same in the two different conditions. The black solid short line, in C, indicates the proliferation ratio of T cells in neat cultures treated with PHA and compared to untreated T cells.

To test our hypothesis that activated B lymphocytes could present antigens to T cells, we isolated B lymphocytes from peripheral lymph nodes of tumor bearing C57Black/6 mice and stimulated the cells with agonist anti-CD40. We observed that the stimulus significantly increased signaling through NFkappaB and ERK, as detected by intracellular staining (ICS) of phosphorylated proteins (Fig 3A). Moreover, *ex vivo* treatment of peripheral lymph node cell suspensions with anti-CD40 led up-regulated expression of CD80, CD86, CD19 and MHC-II, leading to a significantly higher percentage of positive cells for all these markers in stimulated cells compared to controls (Fig 3B). This result is in agreement with our findings about B lymphocytes from cancer patients. Finally, stimulated B lymphocytes from tumor bearing donors could activate T cells also from tumor bearing donors, as shown by proliferation dye dilution (Fig 3C, pep corresponds to cells incubated with HPV peptides) and expression of CD137 and CD154 (Fig 3D). The inhibition of T cells in co-culture with activated B lymphocytes treated with PHA caused an unexpectedly high rate of cell death (probably due to over stimulation [34], which was reflected in the reduction of the proliferation ratio of T cells in this condition compared to T cells in co-culture with control B lymphocytes. As a control for these experiments, we had neat T cells stimulated or not with PHA, with a proliferation ratio of 5.9±0.7 (indicated by the solid black line, Fig 3C).

### Stimulated B lymphocytes, together with T cells from tumor bearing donors, controlled tumor growth in RAG1^-/-^ recipients

**Fig 4:**
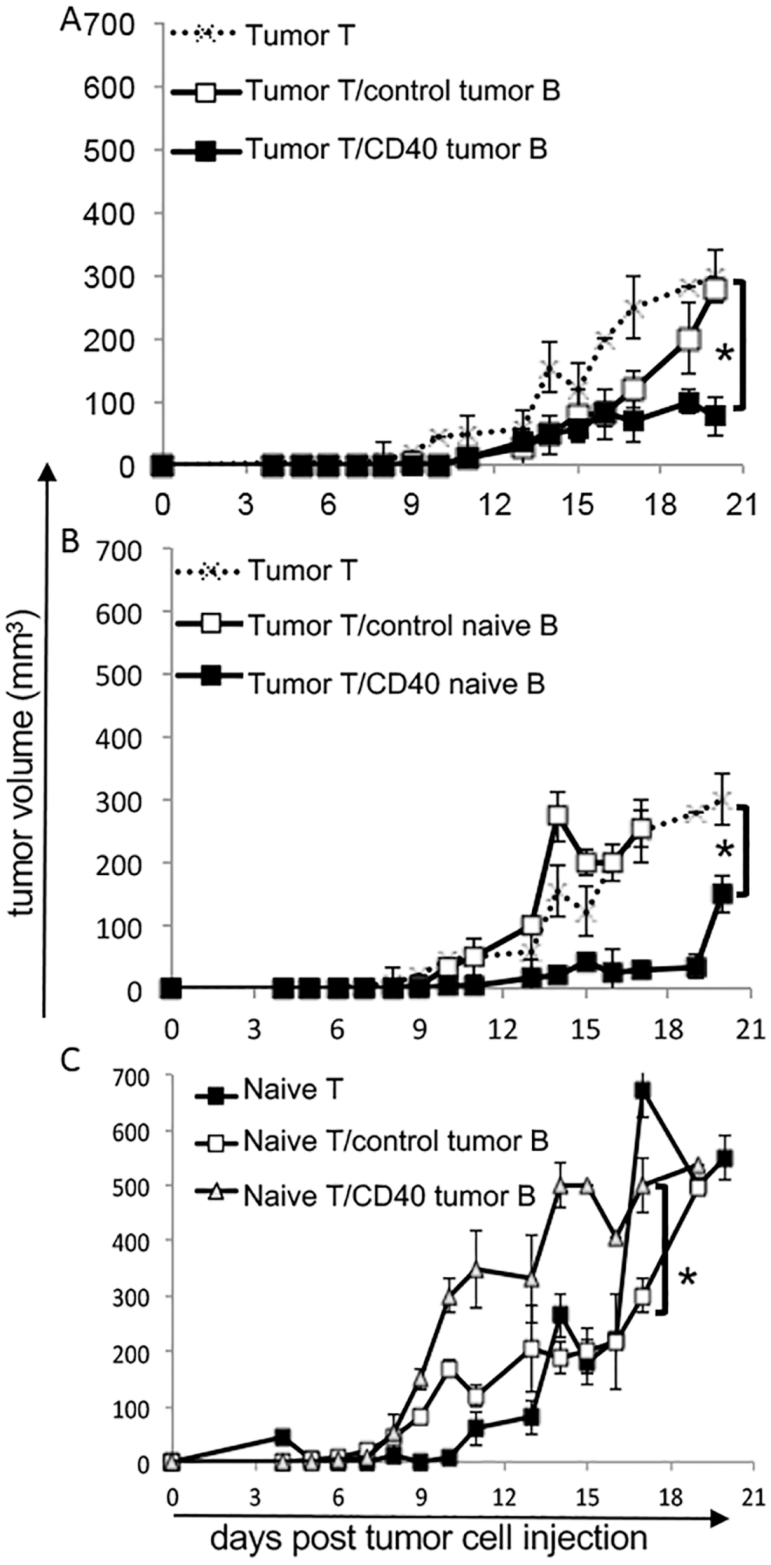
Tumor growth kinetics in mouse chimeras. Lymphocytes isolated from C57Black/6 mice were transplanted into RAG1^-/-^ mice previously injected with 5x10^4^ TC-1 cells. One to 3 million lymphocytes were transplanted per mouse. When mixed, B or DC and T lymphocytes were transplanted in a 1:1 ratio. (A) T lymphocytes from tumor bearing donors, Tumor T, were injected alone or with B lymphocytes from tumor bearing donors activated with anti-CD40 or control: /CD40 tumor B or /control tumor B. (B) T cells from tumor bearing donors, Tumor T, were injected alone or with B cells from naïve donors, activated with anti-CD40 or control: /CD40 naive B or /control naive B. (C) T lymphocytes from naïve donors, Naïve T, were injected into the recipients, alone or with B lymphocytes from tumor bearing donors activated with anti-CD40 or control: /CD40 tumor B and /control tumor B. Experimental groups of 4 to 5 mice (the experiments were repeated 2 times, so a total of at least 8 to 10 mice were tested). Differences between groups was tested by Mann-Whitney U test, the tumor growth kinetics had experimental groups of at least 6 mice; * indicates p<0.05. Significant differences between groups had the same pattern when tested by ANOVA using as data the areas under the curves.

Our results indicated that stimulated B lymphocytes could activate T cells *in vitro*. Therefore, we proceeded to *in vivo* experiments, where we activated isolated B lymphocytes with agonist anti-CD40 antibody, washed the cells thoroughly, mixed them with isolated T cells and immediately transferred all cells to RAG1^-/-^ mice, previously injected with TC-1 tumor cells. Fig 4 shows the different configurations of this experiment. When T and B lymphocytes were transferred from tumor bearing donors, we observed that the combination of activated B lymphocytes with T lymphocytes (Tumor T/CD40 tumor B) significantly inhibited tumor growth compared to control B lymphocytes combined with T lymphocytes (Tumor T/control tumor B), or T lymphocytes alone (Tumor T) (Fig 4A). Interestingly, B lymphocytes from naïve donors, when activated and mixed with T lymphocytes from tumor bearing donors, (Tumor T/CD40 naïve B) also protected mice from tumor growth (Fig 4B). There were no significant differences between tumor growth kinetics of chimeras transplanted with T cells from tumor bearing donors together with activated B cells from naïve or tumor bearing donors (comparison through Mann-Whitney U test and ANOVA using the areas under the curves as data), indicating that previous contact of B lymphocytes with antigens was not necessary for T cell activation. Although, it was not necessary for B lymphocytes to be previously exposed to the tumor antigens, it was imperative for T lymphocytes to be previously primed by tumor antigens. The transfer of T lymphocytes from naïve donors, together with stimulated B, (Naïve T/CD40 tumor B), or non stimulated B lymphocytes, (Naïve T/control tumor B) from tumor bearing donors, significantly increased tumor growth (Fig 4C) (growth similar to tumor growth in non reconstituted RAG1^-/-^ mice, data not shown). Treatment of RAG1^-/-^ mice with anti-CD40 alone had no effect on tumor growth (S4 Fig). However, reconstitution of RAG1^-/-^ mice with T cells from tumor bearing donors together with one dose of anti-CD40, injected directly in the mice, inhibited tumor growth (S4 Fig). This result is somewhat expected since transfer of T cells occurred a few days after tumor cell inoculation in the recipients. At this point, we did not expect any systemic effects of the tumor mass over the immune system. Therefore, myeloid APCs in these RAG1^-/-^ mice could be responsive to the anti-CD40 treatment and present antigens to activate the transferred T cells.

**Fig 5:**
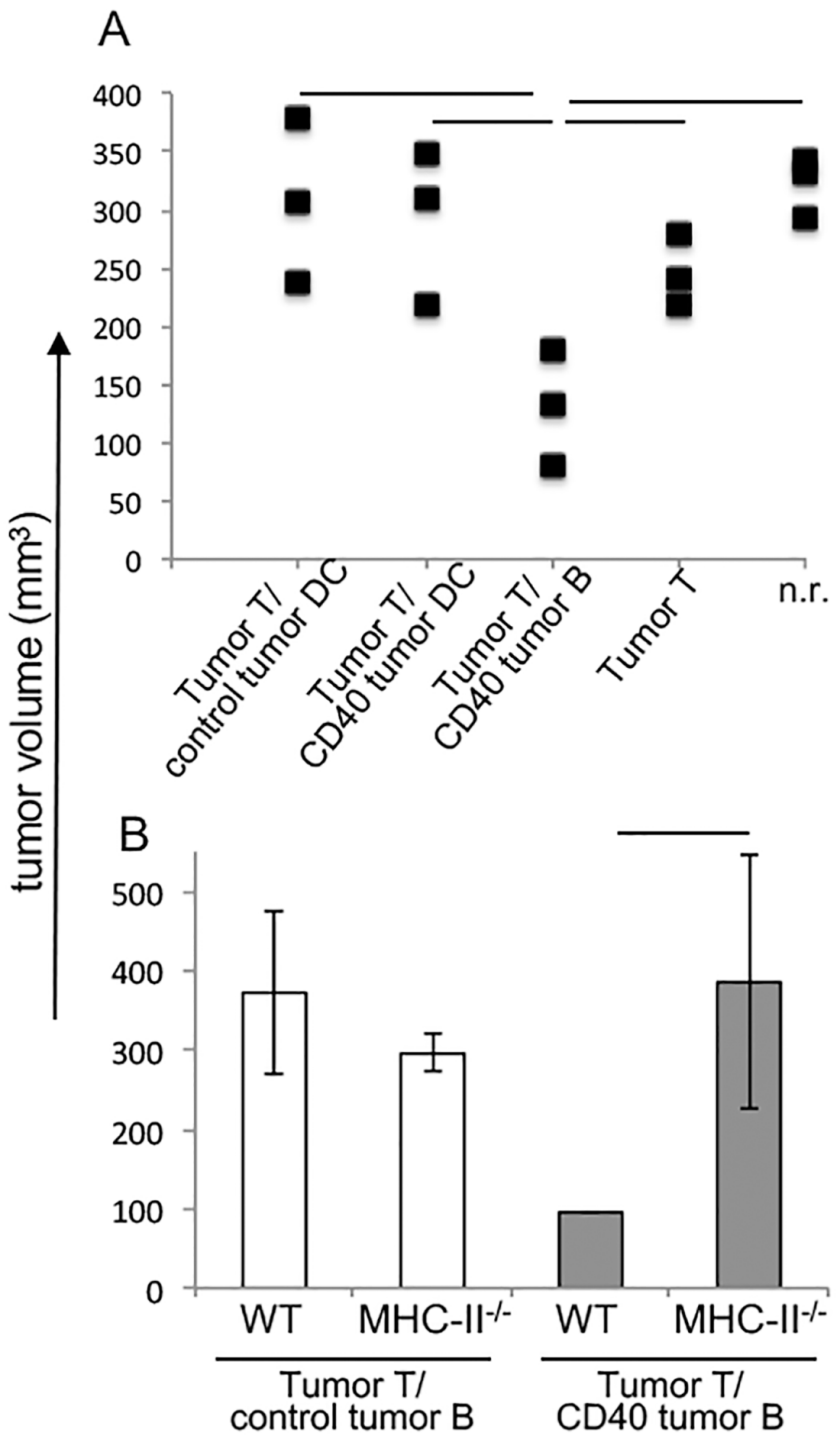
A. Chimeras with dendritic cells, DCs. (A) DCs were isolated from tumor bearing C57Black/6 donors and stimulated or nor with anti-CD40 for 16 hours: /CD40 tumor DC and /control tumor DC, respectively. Cells were washed and mixed with T lymphocytes also from tumor bearing donors, Tumor T, injected into RAG1^-/-^ recipients. As controls, we had a group transplanted with anti-CD40 treated B (Tumor T/CD40 tumor B) lymphocytes and T lymphocytes from tumor bearing donors, a group transplanted with only T lymphocytes (Tumor T) and non reconstituted RAG1^-/-^ group, n.r. (B) Tumor volume in chimeras transplanted with T and B lymphocytes from tumor bearing donors (tumor T and tumor B) that were C57Black/6 (WT) or MHC-II^-/-^. B lymphocytes were treated with anti-CD40 (CD40) or untreated cells (control) (Tumor T/CD40 tumor B and Tumor T/control tumor B). Tumor growth was monitored for 15 to 16 days. Each experimental group had 4 to 6 mice. Differences between groups were tested by ANOVA two way. The tumor growth kinetics had experimental groups of at least 6 mice; * indicates p<0.05.

To test if DCs from our donor mice could activate T cells, we isolated dendritic cells from tumor bearing mice and activated these cells *ex vivo* with anti-CD40, using the same protocol we used for B lymphocytes. Stimulated or control DCs from tumor bearing donors transplanted into RAG1^-/-^ mice together with T cells also from tumor bearing donors were unable to inhibit tumor growth (Tumor T/control tumor DC and Tumor T/CD40 tumor DC) (Fig 5A).

Our working hypothesis was that B lymphocytes could present antigens to T cells and activate secondary responses, inducing an anti-tumor response. If that was the case, tumor growth inhibition, in our system, should be dependent on antigen presentation. Therefore, we repeated the experiment, using B lymphocytes from MHC-II^-/-^ mice, which cannot present antigens through the class II pathway. As shown in Fig 5B, the transfer of activated MHC-II^-/-^ B lymphocytes with T lymphocytes from tumor bearing donors could not inhibit tumor growth in RAG1^-/-^ mice, indicating that the control of tumor growth was dependent on antigen presentation by the B lymphocytes.

**Fig 6:**
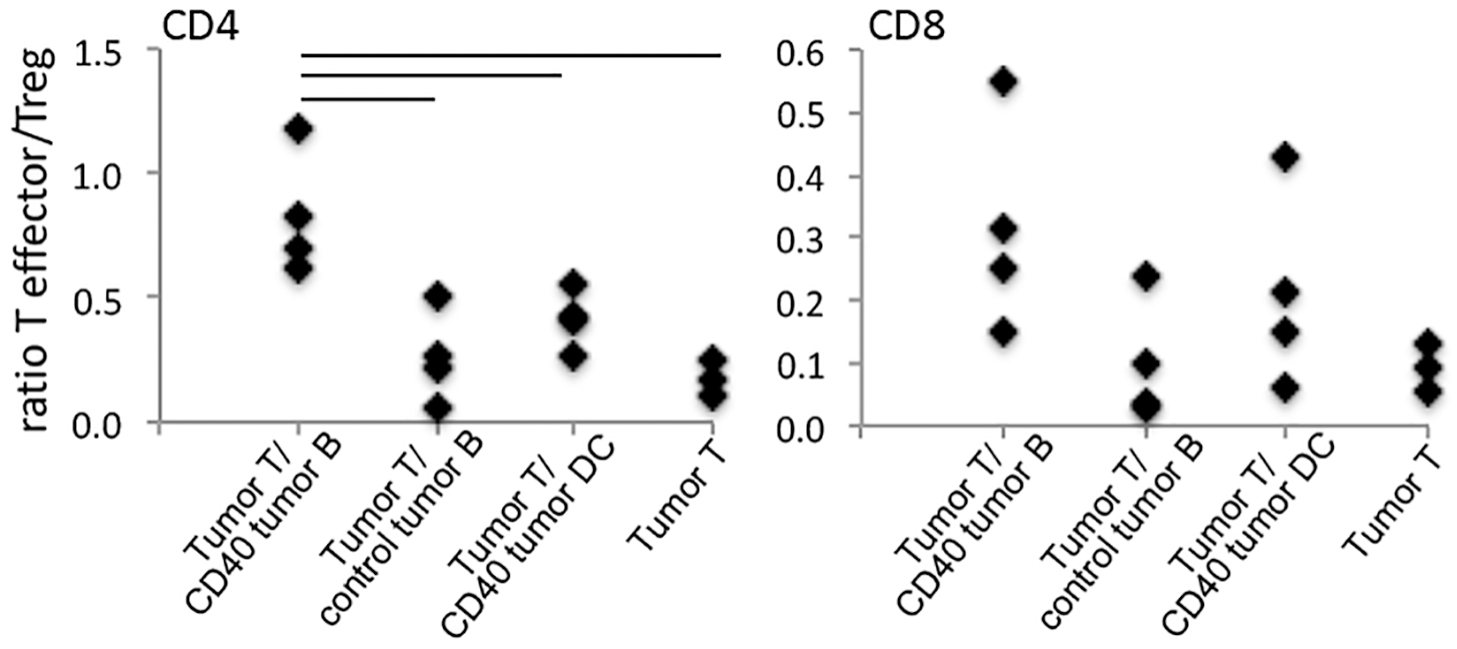
Changes in tumor infiltrating T lymphocyte populations. Flow cytometry analyzes of tumor cell suspensions from chimeras transplanted with T cells from tumor bearing donors alone (Tumor T) or together with anti-CD40 treated or control B lymphocytes from tumor bearing donors (/control Tumor B and /CD40 tumor B, respectively) or with anti-CD40 treated splenic dendritic cells (/CD40 tumor DC). Single cell suspensions were labeled with anti-CD45, CD11b, CD4, CD8, CD25, fixed, permeabilized and labeled with anti-Foxp3 and analyzed by flow cytometry. To identify the lymphocytes, after debris and doublets exclusion, we gated cells in the CD45^+^CD11b^-^ population, then CD4 or CD8 cells, and within the CD4^+^ population, selected CD25^+^Foxp3^+^ cells as regulatory T cells. Data represents the ratio between total CD4 or CD8 T cells and CD4 regulatory T cells. Tumor growth was monitored for 15 to 16 days. Each experimental group had 4 or 5 mice. Data was analyzed by ANOVA; * indicates p values <0.05.

The ratio between conventional T cells and regulatory T cells in the tumor leukocyte infiltrate is associated with anti-tumor immune responses and patients’ prognosis [5, 35,36]. We found that the TILs (Tumor Infiltrating Lymphocytes) from tumors growing in RAG1^-/-^ mice transplanted with activated B cells and T cells from tumor bearing donors had a significantly higher conventional/regulatory T cell ratio than tumors from the other experimental groups (Fig 6). This effect was not observed with the CD8 population, likely due to the somewhat high experimental variability.

## Discussion

Many years ago, Doan and collaborators demonstrated, in a transgenic experimental model, that there was peripheral tolerance toward HPV antigens [37]. Later, van der Burg and collaborators also showed evidence of peripheral tolerance in cervical cancer patients [6]. Several research groups have been investigating the mechanisms underlying the tolerance toward HPV antigens, including ours [25,29]. In this manuscript, we have shown, that simply removing T lymphocytes from the tumor bearing host and transferring them to another host, with established but undetectable tumors to the naked eye and touch, allowed these cells to display anti-tumor activity. This result indicated, first, that T cells from tumor bearing mice had the potential to recognize and respond to tumor antigens; second, that the tumor induced a tolerogenic environment in the lymph nodes and spleen, therefore causing systemic effects on the immune system.

We had previously shown, using both the TC-1 tumor model injected into C57Black/6 immunocompetent mice [25], or cervical cancer derived cell lines, SiHa, HeLa and C33A injected into Nude or RAG1^-/-^ immunodeficient mice [38], that HPV associated tumors induced proliferation of myeloid cells in the bone marrow and spleen, which had tolerogenic activity, suppressing T cells [29]. Others have shown that a significant percentage of the cervical cancer patients display leukocytosis, mainly due to the increase of myeloid cells in the circulation, which was linked to the secretion of G-CSF by tumor cells [39]. Importantly, leukocytosis is a poor prognostic factor for cervical cancer patients [40]. The role of immunosuppression of myeloid cells in cancer patients and experimental models has been amply demonstrated in the literature [41] In cervical cancer patients, we have shown that monocyte derived DCs inhibit T cell activation. The same was not observed if monocytes were harvested from patients with cervicitis or cervical precursor lesions [16], indicating that only advanced tumors were capable of creating a systemic tolerogenic effect. In our experimental model, we have shown that very small tumors in RAG1^-/-^ mice did not affect APCs, since a single injection of anti-CD40, together with T cell transfer from tumor bearing donors, could protect mice from tumor growth. This result, guarding the differences between the two experimental settings, confirmed that only larger or more advanced tumors caused systemic effects on the host’s immune system.

The premise of our work is that B lymphocytes would be more resilient to the systemic effects of the tumors and would be able to present antigens to T cells. As mentioned before, B lymphocytes are mostly considered a pro-tumoral population. Inhibition of B lymphocyte activation with Ibrutinib, for example, augments anti-tumor responses [19,22,23]. Interestingly, it has been shown that myeloid derived suppressor cells (MDSC) and other cells in the microenvironment also signal through BTK, the kinase inhibited by Ibrutinib, and the inhibition of this signaling pathway may enhance anti-tumor T cell responses, not only due to inhibition of B lymphocytes, but also other regulatory populations [42,43]. Moreover, there is direct evidence that properly activated B lymphocytes may induce anti-tumor T cell responses [44]. As mentioned before, B lymphocytes from cervical cancer patients were able to present antigens to T cells in *ex vivo* assays [6].

In our experimental model, we observed that B lymphocyte deficiency had no effect on tumor growth, contrary to the absence of T cells. The role of T cells in controlling HPV associated tumor growth was clearly observed by research showing that HIV infected immunosuppressed patients displayed higher risk of developing cervical cancer than controls [45].

In human cells, best activation of B cells was achieved by combining soluble CD40 with IL-4. The same experimental conditions could also activate for B lymphocytes from cancer patients. In the mouse, we found that the treatment with anti-CD40 alone displayed significant results on B cell activation and decided to work with a single stimulus in the experimental model. CD40 signals through NFkappaB, MAPK, PI3K, among other pathways leading to cytokine expression, co-stimulatory molecules expression, cell proliferation and survival and the obvious functions of B lymphocytes, such as isotype switching and antibody production [46,47]. In our case, anti-CD40 treatment activated both NFkappaB and MAPK (ERK), which are typically involved in the expression of co-stimulatory molecules and B cell proliferation and activity. Our results indicated that anti-CD40 could stimulate B lymphocytes from tumor bearing mice, which led to an increase in the expression of proteins in the antigen presentation machinery and activation of secondary responses in T lymphocytes previously exposed to tumor antigens. *In vitro*, we observed that activated B lymphocytes could induce antigen specific T cell proliferation and expression of activation markers.

B lymphocytes from tumor bearing or naïve donors had similar effects in inhibiting tumor growth when transplanted together with T cells, which was intriguing and led us to hypothesize that these cells may be capturing antigens *in vivo* and presenting to T cells after transplant to the recipient mice. This was a reasonable hypothesis, since we have observed that antigen presenting cells in RAG1^-/-^ mice could be activated by injection of anti-CD40 and activate transferred T lymphocytes from tumor bearing donors. Of notice this was not tested in later time points than at the first detection of tumors. We expect that, as tumor grew larger the myeloid APCs in the RAG1^-/-^ mice would be modulated by the tumor and inhibit T cells. Under another perspective, the donor cells used in our study were harvested at day 21 post TC-1 tumor cells injection into wild type mice. Therefore, at this time point, whatever systemic effect these tumors would have over the immune system would be at work. Indeed, anti-CD40 treated DCs isolated from donors with TC-1 tumors could not inhibit tumor growth in RAG1^-/-^ mice when transplanted with T cells, differently from what we observed with the B lymphocytes, which suggested that these cells were more resistant to the effects of the tumor. In accordance to that, our laboratory has previously shown that CD11b^+^ splenocytes from TC-1 tumor bearing mice induced regulatory phenotype in T cells in *in vitro* assays [29]. These results corroborate the inhibition of allogeneic T cell activation by monocyte derived dendritic cells from cervical cancer patients, even after cytokine activation [16].

Another interesting observation in our work was that it was essential that T lymphocytes were previously exposed to tumor antigens for protection against tumor growth. If we used T cells from naïve donors, the results were the opposite. Indeed, it has been demonstrated, for many years, that B lymphocytes tolerize naïve T lymphocytes, except under special conditions [48,49], probably to avoid immune responses to auto-antigens.

Regarding tumor antigen specific responses in our experimental model, we are suggesting that T cell responses triggered by the activated T lymphocytes are specific to tumor or HPV antigens. We have not been able to actually prove that *in vivo*. It is a fact that, tumor growth inhibition depends on MHC-II expression by B lymphocytes, however, we have not actually determined which antigens were presented to T cells. We showed that activated B cells could increase specific antigen driven T cell proliferation *ex vivo*, but not *in vivo* (for example, by using MHC tetramers with tumor epitopes). Experimental data from other research groups have shown that patients actually mount antigen specific T cell responses against HPV associated tumors that are suppressed in the tumor microenvironment. They also showed that immunomodulators, such as ligands to Toll like Receptors can increase these responses [7,32]. Moreover, we have proved that the immune system of C57Black/6 mice mounts specific T cell responses against TC-1 tumors [25,29], so that it is possible that the inhibition of tumor growth in our chimeras, is driven by B cell induced antigen specific T cells responses.

Our data suggest that B lymphocytes may be considered as a tool to promote anti-tumor T cell responses, if properly stimulated. It would be necessary to combine ablative treatments as chemotherapy or radiotherapy previously to lymphocyte transfer, in order to release tumor antigens, and create proper niches for T cell expansion.

## Supporting information

S1 Fig Optimization of human B lymphocytes activation. B lymphocytes from healthy donors were stimulated for the indicated periods of time with 0, 0.1, 1 or 10 μg/ml sCD40L, after which labeled cells with anti-CD80, anti-CD86 and anti-HLA-DR and evaluated cells by cell cytometry (top panels). The same kind of experiment was performed with addition of 20 ng/ml IL-4 to the cells (bottom panels). Data is represented by the frequency of CD80^+^CD86^+^ cells and HLA-DR expression (MFI, median of fluorescence intensity).
(PDF)

S2 Fig Circulating B cells in invasive cancer patients and control subjects. PBMCs isolated from both invasive cervical cancer patients or age matched control subjects were labeled with anti-CD19, anti-CD38, anti-CD21, anti-CD27, anti-IgD, anti-IgM and analyzed by flow cytometry. The graphs show the percentage of CD19^+^ in the total population, and the percentage of naïve and memory cells within the CD19^+^ population. Naïve phenotype was defined as CD19^+^CD27^-^IgD^+^ and memory as CD19^+^CD27^+^IgD^-^CD38^-/+^.
(PDF)

S3 Fig T cells are the main population that respond to TC-1 tumors. C57Black/6 (C57), BKO and RAG1^-/-^ (RAG) mice were injected with 1.5x10^4^ TC-1 cells subcutaneously and tumor growth was followed as indicated. The graph shows tumor volume increase through time. Differences between groups was tested by Mann-Whitney U test and ANOVA with the data derived from the areas under the curves; experimental groups of 5 or 6 mice; * indicates p<0.05.
(PDF)

S4 Fig Controls for tumor growth kinetics in mouse chimeras. Lymphocytes isolated from C57Black/6 mice were transplanted into RAG1^-/-^ mice previously injected with 5x10^4^ TC-1 cells. One to 3 million lymphocytes were transplanted per mouse as follows: T cells from naïve or tumor bearing donors alone (Naïve T and Tumor T, respectively), Tumor T cells and one dose of 10μg of anti-CD40 (Tumor T/CD40) and an injection of anti-CD40 alone (CD40). Differences between groups was tested by Mann-Whitney U test; the tumor growth kinetics had experimental groups of at least 6 mice; * indicates p<0.05.
(PDF)
