# Supplementary material for: B lymphocytes can be activated to act as antigen presenting cells to promote anti-tumor responses
Source: PLoS One. 2018 Jul 5;13(7):e0199034. doi: 10.1371/journal.pone.0199034 (PMC6033398; doi:10.1371/journal.pone.0199034)
Supplement: S1 Fig — B lymphocytes from healthy donors were stimulated for the indicated periods of time with 0, 0.1, 1 or 10 μg/ml sCD40L, after which labeled cells with anti-CD80, anti-CD86 and anti-HLA-DR and evaluated cells by cell cytometry (top panels). The same kind of experiment was performed with addition of 20 ng/ml IL-4 to the cells (bottom panels). Data is represented by the frequency of CD80+CD86+ cells and HLA-DR expression (MFI, median of fluorescence intensity). (PDF) [file pone.0199034.s001.pdf]

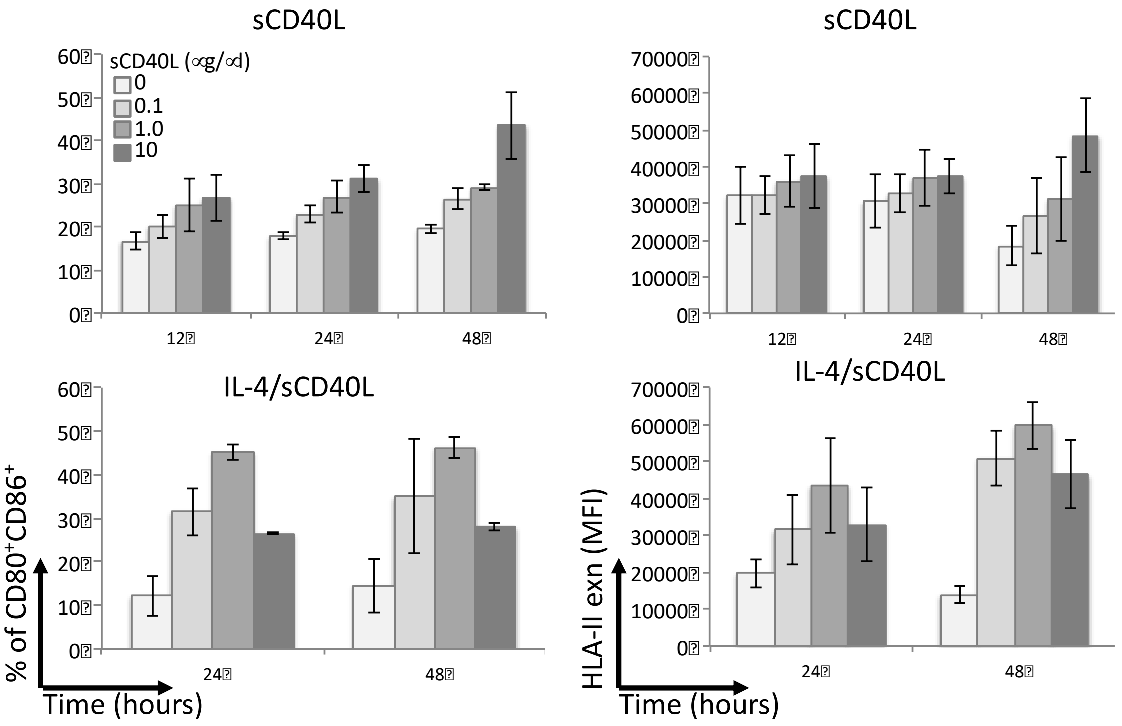

S1 Fig. Optimization of human B lymphocytes activation. B lymphocytes from healthy donors were stimulated for the indicated periods of time with 0, 0.1, 1 or 10 μg/ml sCD40L, after which labeled cells with anti-CD80, anti-CD86 and anti-HLA-DR and evaluated cells by cell cytometry (top panels). The same kind of experiment was performed with addition of 20 ng/ml IL-4 to the cells (bottom panels). Data is represented by the frequency of $CD80^{+}CD86^{+}$ cells and HLA-DR expression (MFI, median of fluorescence intensity).
