# Supplementary material for: B lymphocytes can be activated to act as antigen presenting cells to promote anti-tumor responses
Source: PLoS One. 2018 Jul 5;13(7):e0199034. doi: 10.1371/journal.pone.0199034 (PMC6033398; doi:10.1371/journal.pone.0199034)
Supplement: S2 Fig — PBMCs isolated from both invasive cervical cancer patients or age matched control subjects were labeled with anti-CD19, anti-CD38, anti-CD21, anti-CD27, anti-IgD, anti-IgM and analyzed by flow cytometry. The graphs show the percentage of CD19+ in the total population, and the percentage of naïve and memory cells within the CD19+ population. Naïve phenotype was defined as CD19+CD27-IgD+ and memory as CD19+CD27+IgD-CD38-/+. (PDF) [file pone.0199034.s002.pdf]

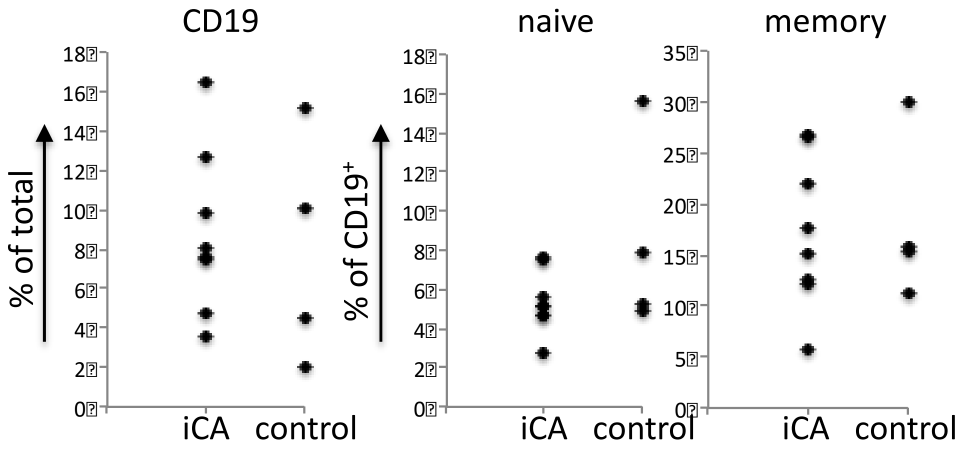

S2 Fig. Circulating B cells in invasive cancer patients and control subjects. PBMCs isolated from both invasive cervical cancer patients or age matched control subjects were labeled with anti-CD19, anti-CD38, anti-CD21, anti-CD27, anti-IgD, anti-IgM and analyzed by flow cytometry. The graphs show the percentage of CD19+ in the total population, and the percentage of naïve and memory cells within the the $CD19_+$ population. Naïve phenotype was defined as $CD19^+CD27^-IgD^+$ and memory as $CD19^+CD27^+IgD^-CD38^{-/+}$.
