# Supplementary material for: B lymphocytes can be activated to act as antigen presenting cells to promote anti-tumor responses
Source: PLoS One. 2018 Jul 5;13(7):e0199034. doi: 10.1371/journal.pone.0199034 (PMC6033398; doi:10.1371/journal.pone.0199034)
Supplement: S3 Fig — C57Black/6 (C57), BKO and RAG1-/- (RAG) mice were injected with 1.5x10^4^ TC-1 cells subcutaneously and tumor growth was followed as indicated. The graph shows tumor volume increase through time. Differences between groups was tested by Mann-Whitney U test and ANOVA with the data derived from the areas under the curves; experimental groups of 5 or 6 mice; * indicates p<0.05. (PDF) [file pone.0199034.s003.pdf]

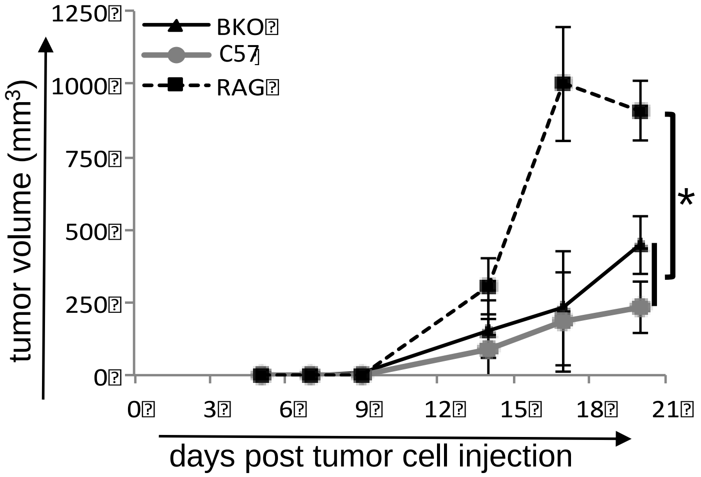

S3 Fig. T cells are the main population that respond to TC-1 tumors. C57Black/6 (C57), BKO and RAG1$^{-/-}$ (RAG) mice were injected with $1.5x10^4$ TC-1 cells subcutaneously and tumor growth was followed as indicated. The graph shows tumor volume increase through time. Differences between groups was tested by Mann-Whitney U test and ANOVA with the data derived from the areas under the curves; experimental groups of 5 or 6 mice; * indicates $p<0.05$.
