# Supplementary material for: B lymphocytes can be activated to act as antigen presenting cells to promote anti-tumor responses
Source: PLoS One. 2018 Jul 5;13(7):e0199034. doi: 10.1371/journal.pone.0199034 (PMC6033398; doi:10.1371/journal.pone.0199034)
Supplement: S4 Fig — Lymphocytes isolated from C57Black/6 mice were transplanted into RAG1-/- mice previously injected with 5x10^4^ TC-1 cells. One to 3 million lymphocytes were transplanted per mouse as follows: T cells from naïve or tumor bearing donors alone (Naïve T and Tumor T, respectively), Tumor T cells and one dose of 10μg of anti-CD40 (Tumor T/CD40) and an injection of anti-CD40 alone (CD40). Differences between groups was tested by Mann-Whitney U test; the tumor growth kinetics had experimental groups of at least 6 mice; * indicates p<0.05. (PDF) [file pone.0199034.s004.pdf]

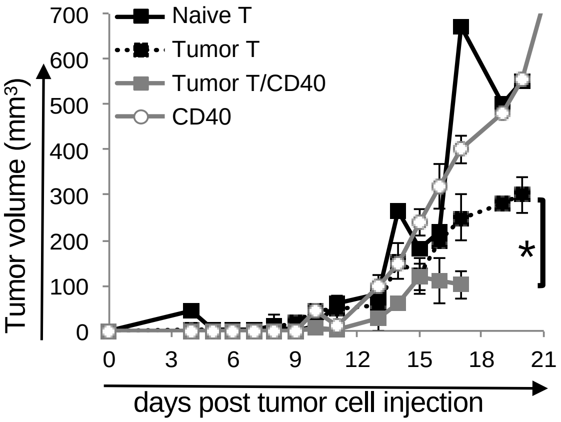

S4 Fig. Controls for tumor growth kinetics in mouse chimeras. Lymphocytes isolated from C57Black/6 mice were transplanted into RAG1$^{-/-}$ mice previously injected with 5x10^4 TC-1 cells. One to 3 million lymphocytes were transplanted per mouse as follows: T cells from naïve or tumor bearing donors alone (Naïve T and Tumor T, respectively), Tumor T cells and one dose of 10µg of anti-CD40 (Tumor T/CD40) and an injection of anti-CD40 alone (CD40). Differences between groups was tested by Mann- Whitney U test; the tumor growth kinetics had experimental groups of at least 6 mice; * indicates $p<0.05$.
